# Supplementary material for: Causal Relationship between Aging and Anorexia Nervosa: A White-Matter-Microstructure-Mediated Mendelian Randomization Analysis
Source: Biomedicines. 2024 Aug 16;12(8):1874. doi: 10.3390/biomedicines12081874 (PMC11351342; doi:10.3390/biomedicines12081874)
Supplement: Supplementary file 1 [file biomedicines-12-01874-s001.zip › Supplementary Figure/Figure S4/Singe SNP plot of MR analysis for TL to ubm-b-1693.pdf]

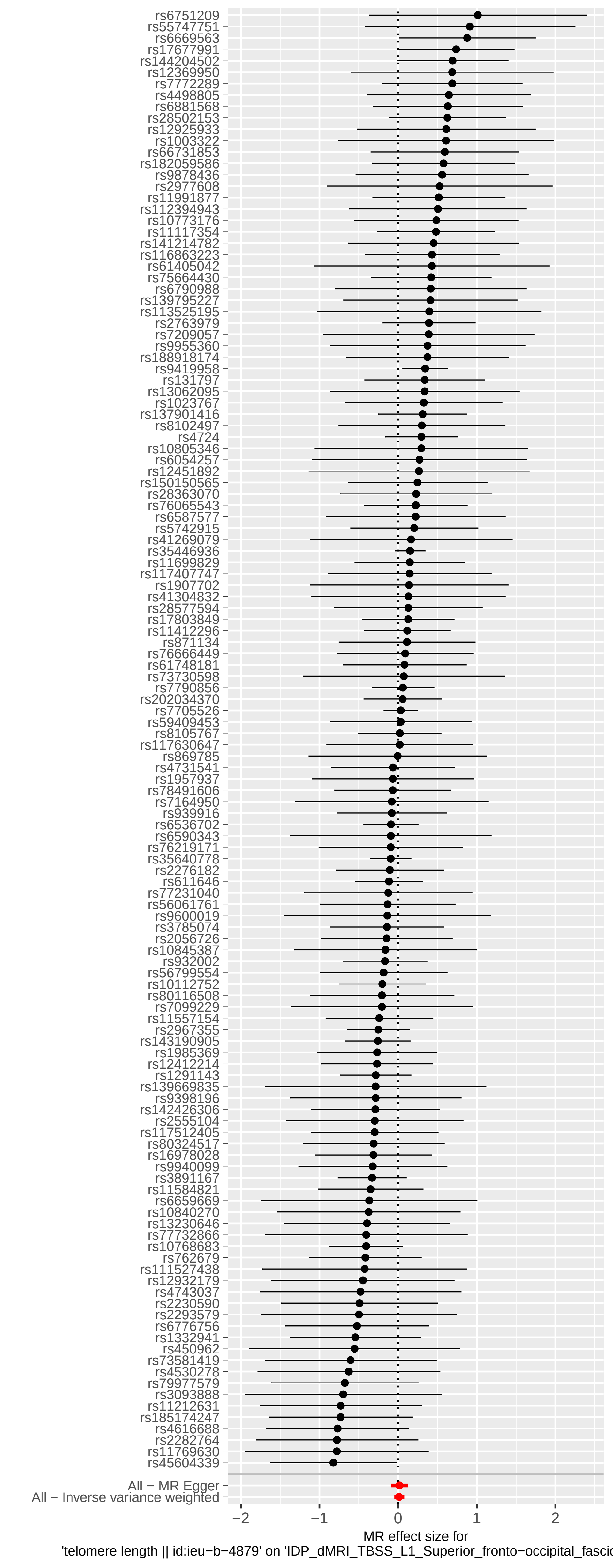

rs6751209
rs55747751
rs6669563
rs17677991
rs144204502
rs12369950
rs7772289
rs4498805
rs6881568
rs28502153
rs12925933
rs1003322
rs66731853
rs182059586
rs9878436
rs2977608
rs11991877
rs112394943
rs10773176
rs11117354
rs141214782
rs116863223
rs61405042
rs75664430
rs6790988
rs139795227
rs113525195
rs2763979
rs7209057
rs9955360
rs188918174
rs9419958
rs131797
rs13062095
rs1023767
rs137901416
rs8102497
rs4724
rs10805346
rs6054257
rs12451892
rs150150565
rs28363070
rs76065543
rs6587577
rs5742915
rs41269079
rs35446936
rs11699829
rs117407747
rs1907702
rs41304832
rs28577594
rs17803849
rs11412296
rs871134
rs76666449
rs61748181
rs73730598
rs7790856
rs202034370
rs7705526
rs59409453
rs8105767
rs117630647
rs869785
rs4731541
rs1957937
rs78491606
rs7164950
rs939916
rs6536702
rs6590343
rs76219171
rs35640778
rs2276182
rs611646
rs77231040
rs56061761
rs9600019
rs3785074
rs2056726
rs10845387
rs932002
rs56799554
rs10112752
rs80116508
rs7099229
rs11557154
rs2967355
rs143190905
rs1985369
rs12412214
rs1291143
rs139669835
rs9398196
rs142426306
rs2555104
rs117512405
rs80324517
rs16978028
rs9940099
rs3891167
rs11584821
rs6659669
rs10840270
rs13230646
rs77732866
rs10768683
rs762679
rs111527438
rs12932179
rs4743037
rs2230590
rs2293579
rs6776756
rs1332941
rs450962
rs73581419
rs4530278
rs79977579
rs3093888
rs11212631
rs185174247
rs4616688
rs2282764
rs11769630
rs45604339
All − MR Egger
All − Inverse variance weighted
−2
−1
0
1
2
MR effect size for
'telomere length || id:ieu−b−4879' on 'IDP_dMRI_TBSS_L1_Superior_fronto−occipital_fascic
